# Ectopic pancreas tissue appearing in a mediastinal cyst

**DOI:** 10.1186/1749-8090-7-22

**Published:** 2012-03-13

**Authors:** Sandor Szabados, László Lénárd, Tamás Tornóczky, Edit Várady, Zsófia Verzár

**Affiliations:** 1 Heart Clinic, University of Pécs, Pécs, Hungary; 2 Department of Anesthesiology and Intensive Care, Faculty of Medicine, University of Pécs, Pécs Hungary; 3 Medical University of Pécs, Department of Heart Surgery Head of the Department, 7624 Pécs Ifjuság street 13. Hungary

## Abstract

Heterotopia of pancreatic tissue is a common developmental anomaly. Although ectopic pancreatic tissue is mostly found in the gastrointestinal tract, localization in the mediastinum is extremely rare. We report a 32-year-old male patient who had an urgent thoracotomy two years ago due to a thoracic surgery. During the thoracotomy fragments of a partly necrotic cystic mass in the right thorax were removed and decortication was performed. Two years later the patient was hospitalized again because of haemoptoe and atypical chest pain. A residual cystic mass was detected between the right hilum and the ascending aorta connecting to the pericardium, the superior vena cava and the aorta on the chest CT. After the operation a mediastinal cyst was diagnosed, with a pancreatic tissue by histology.

## Case report

**Figure 1:**
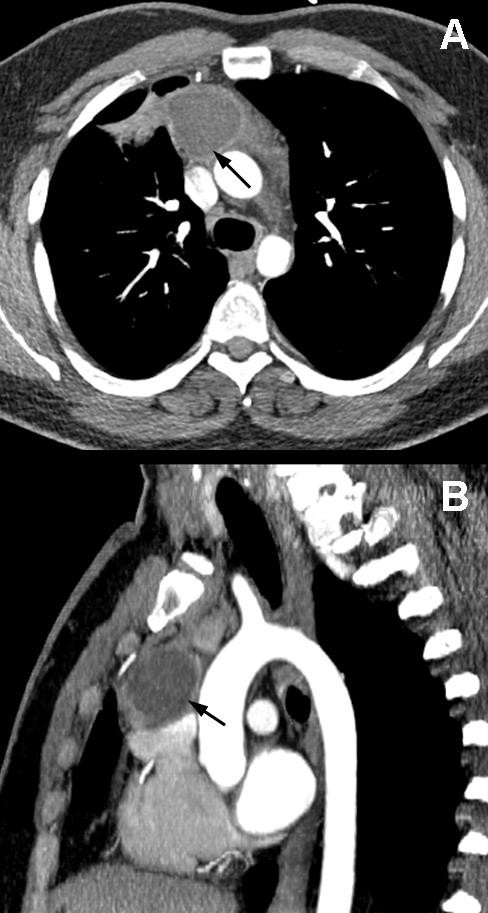
**Chest CT -The arrows show the residual cystic mass detected between the right hilum and the ascending aorta connecting to the pericardium, the superior vena cava and the aorta on chest CT (A,B)**.

A 32 years old male had thoracotomy two years earlier due to an intra-thoracic pathology. Before that operation the lesion was thought to be lymphoma based on the enlarged Mediastinal lymph nodes and fever. A bone marrow biopsy was negative. Progression was Detected in the clinical status and pneumonia with a large pleural effusion and sepsis Developed. Thoracocentesis yielded 200 ml turbid fluid which was sent to cytological and Microbiological examinatiopns, all of which were negative. The condition of the patient with Antibiotic therapy failed to improve, therefore an urgent thoracotomy was done. Fragments of A partly necrotic cystic mass from the right thorax were removed and decortication Perforemed. The postoperative period was uneventful. The patient was discharged on the 6ht Postoperative day. However, he failed to return for follow up. Two years later he was Hospitalized again because of hemoptysis and chest pain. A cystic mass was detected between The right hilum ascending aorta, connected to the pericardium, the superior vena cava and the Aorta. (Chest CT, Figure 1.)

**Figure 2:**
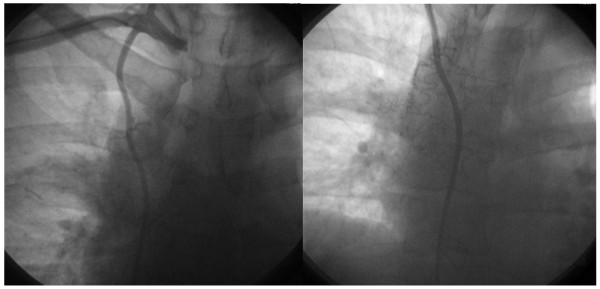
**Angiography - showed some blood supply of the cyst from the right internal thoracic artery but not from the coronary arteries**.

**Figure 3:**
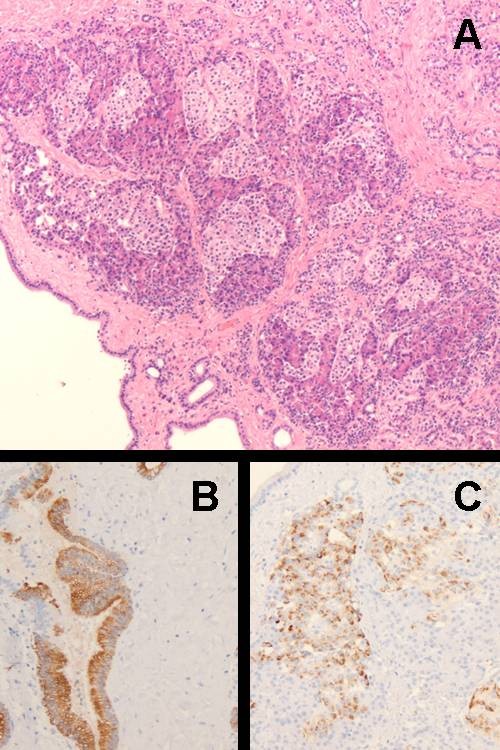
**HE-stained sections made of the specimens showed mature pancreas tissue within the wall of the cyst (A)**. Immunohistochemistry highlighted the cytokeratin positive ducts and chromogranin positive Langerhans inslets (B,C).

Angiography showed no coronary involvement and blood supply to the mass from the right Internal mammary artery (RIMA). (Figure 2) At the median sternotomy the entire lesion with the Involved portion of the pericardium was removed. HE-staied ection of the specimen showed Mature pancreatic tissue in the wall of the cyst. (Figure 3A) Immunhistochemistry highlighted

The cytokeratin-positive ducts (Figure 3B) and chromogranin-positive Langerhans islets. (Figure 3C)

The patient was discharged without any complications.

Ectopic pancreas tissue may appear in this region, therefore it should be include in the Differential diagnosis of the mediastinal cyst.

## Comment

Ectopic pancreatic tissue is a common anomaly, reported in about 2% of autopsies [1,2]. This Tissue has been found most commonly in the stomach, duodenum, jejunum or ileum [3,4].

Mediastinal cyst formed by pancreatic tissue is extremly rare [5]. We found only six cases in The literature [6]. We report ours because it was recurrent - the tissue diagnosis missed at the First resection - and was attached to the pericardium. Since the total exstiraptin of the cyst two Years ago, the patient remained asymptomatic.

## Consent

Written informed consent was obtained from the patient for publication of this Case report and any accompanying images. A copy of the written consent is available for review by the Editor-in-Chief of this journal.

## Competing interests

The authors declare that they have no competing interests.

## Authors' contributions

In the following we specify the individual contributions of authors to the manuscript. Ssz and LL operated the patient, TT made the histological examinations, EV made the CT examinations zsv prepared the manuscript. All authors read and approved the final manuscript.
